# Supplementary figures and images for: A leaky gut dysregulates gene networks in the brain associated with immune activation, oxidative stress, and myelination in a mouse model of colitis
Source: bioRxiv. 2023 Aug 13:2023.08.10.552488. Preprint. [Version 1] doi: 10.1101/2023.08.10.552488 (PMC10441416; doi:10.1101/2023.08.10.552488)

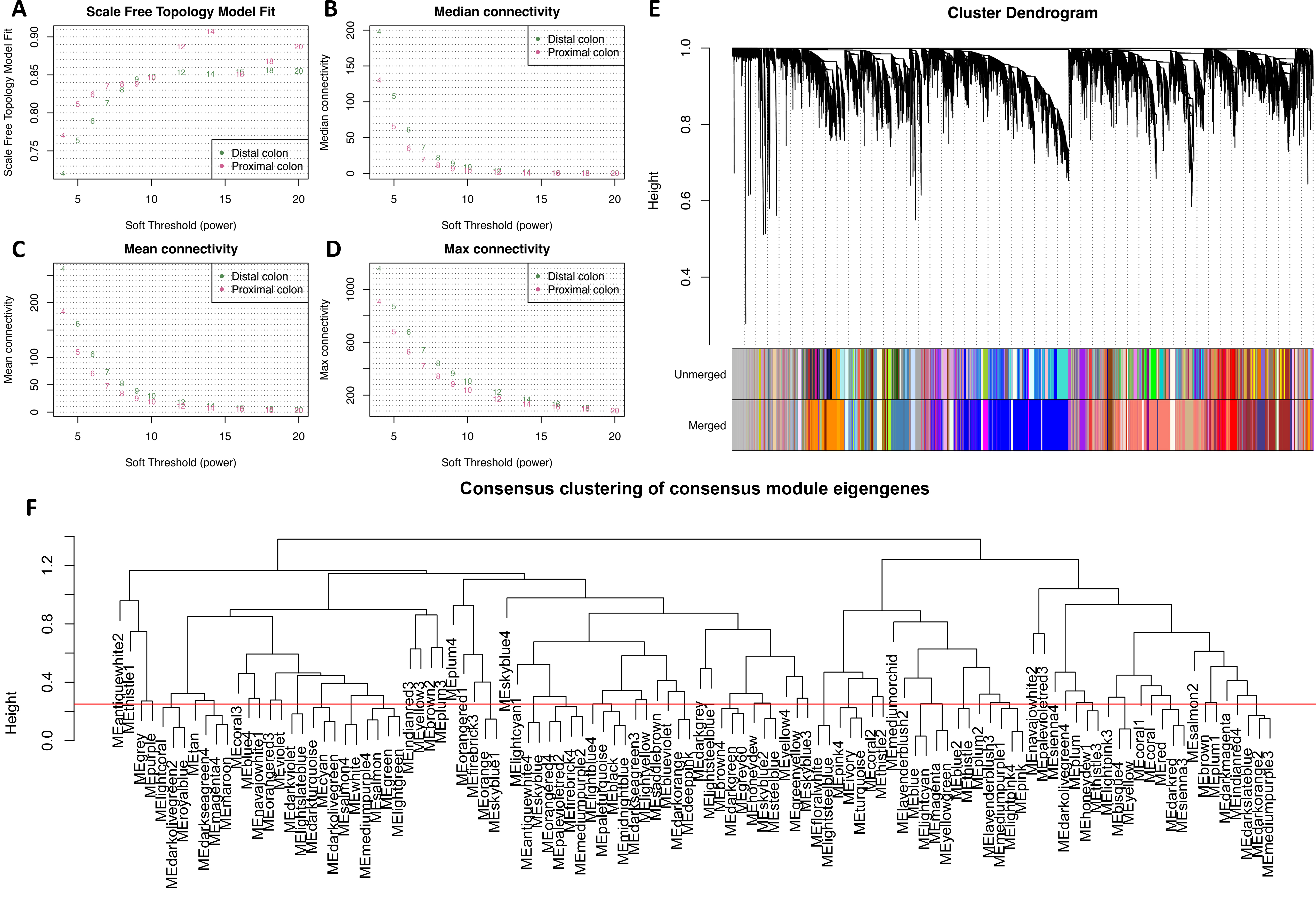

Supplement: Supplement 1 — Supp. Figure 1: Consensus co-expression network construction in two colon segments. A Scale-free topology model R2 in distal (green) and proximal (pink) colon segment RNA sequencing datasets as a function of soft thresholding/beta power. The median (B), average (C), and maximum (D) connectivity of both segments are shown as functions of soft thresholding power. From these data, a soft power of 7 was selected to reach a model fit above the recommended threshold of 0.8, which is also the point at which connectivity does not decrease substantially as soft threshold power increases. E Gene dendrogram with original (unmerged) and merged module labels. Modules were merged based on average linkage distance shown in the dendrogram in F. [file media-1.jpg]

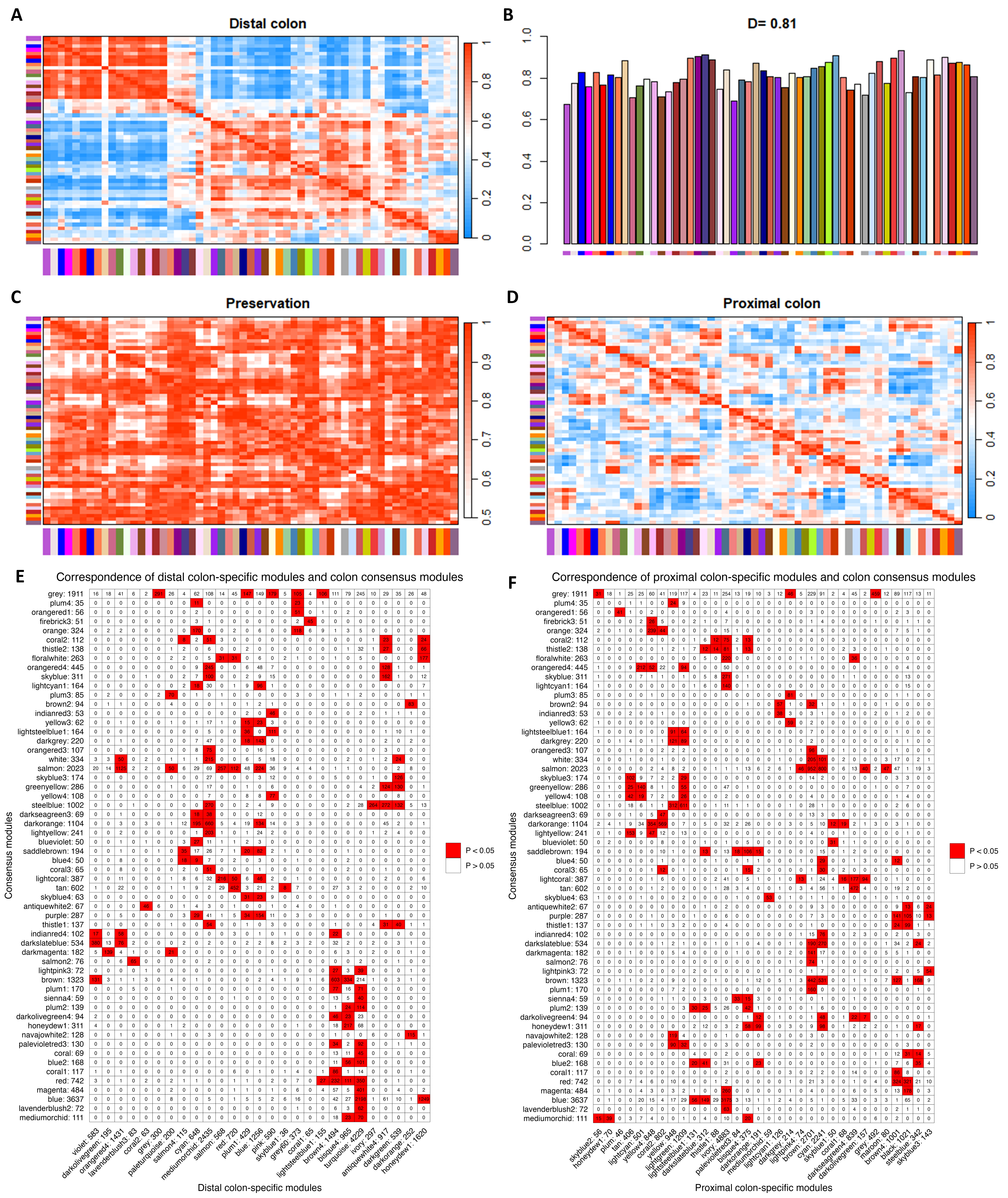

Supplement: Supplement 2 — Supp. Figure 2: Assessment of gene network preservation between distal and proximal colon segments. A Intermodular correlation heatmap in distal colon. Each row and column correspond to the eigengene of the module. Red represents a positive correlation between modules, while blue represents a negative correlation. B Bar chart showing the degree of preservation of each consensus module in both colon networks, where taller bars indicate a greater degree of preservation. The overall high D-value shown at the top indicates a strong preservation of modules between both networks. C Heatmap displaying the difference between networks in eigengene adjacencies. Preservation is calculated as one minus the absolute value of the difference of the eigengene correlations in the two colon networks. Thus, a brighter red indicates a smaller difference between networks. D Intermodular correlation heatmap in proximal colon, coded the same as A. E Multiple Fisher’s exact test comparing the independence of gene assignment in distal colon-specific modules and gene assignment to consensus modules. Rows are consensus modules and columns are distal colon-specific modules. Cells shaded red indicate a significant Fisher’s exact test (p < 0.05) after Bonferroni’s correction, meaning there is significant overlap between the distal colon-specific module and the consensus module. The number in each cell reflects the number of genes overlapping the two modules shown at that intersection. F Multiple Fisher’s exact test comparing the independence of gene assignment in proximal colon-specific modules and gene assignment to consensus modules, coded the same as in E. [file media-2.jpg]

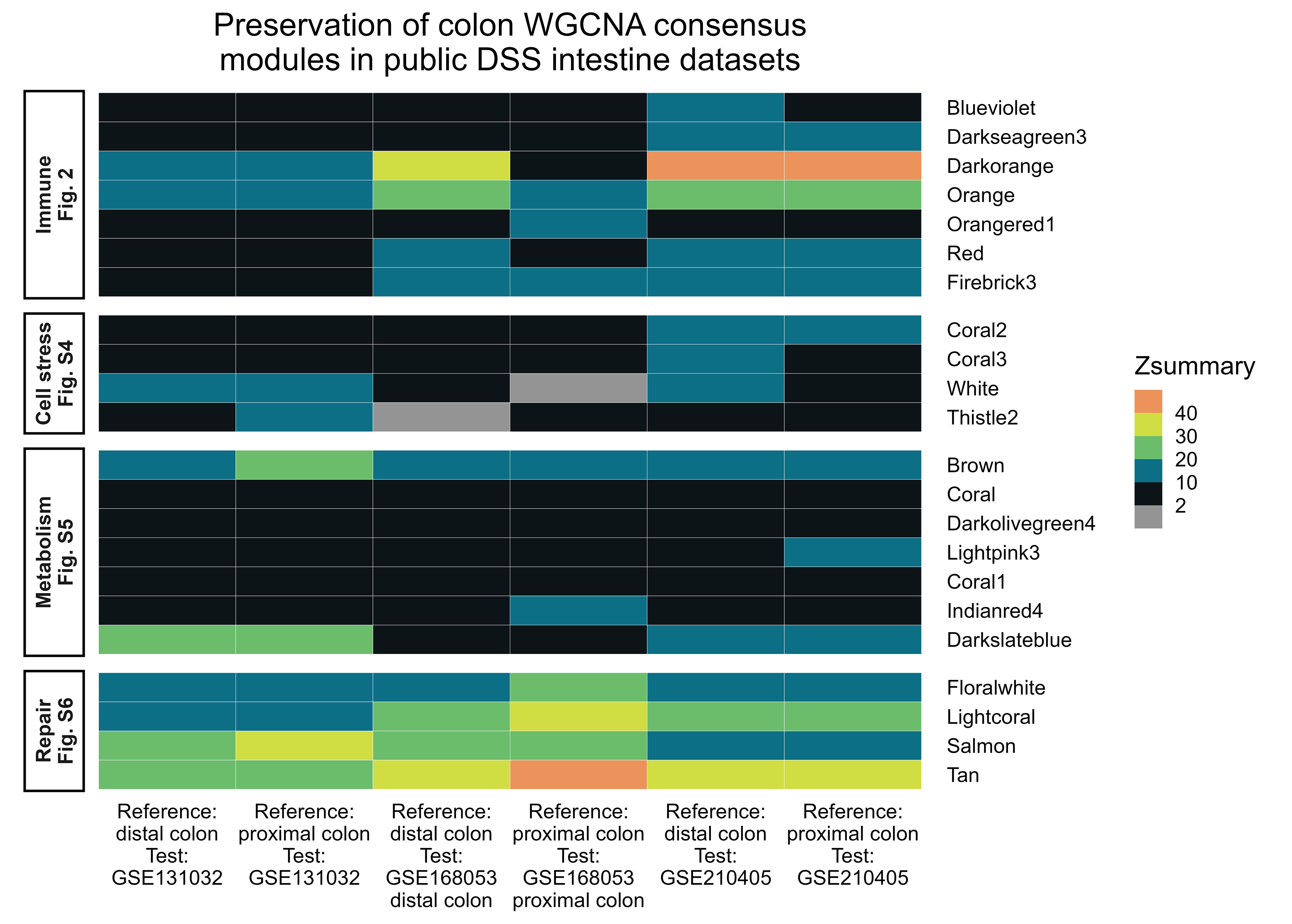

Supplement: Supplement 3 — Supp. Figure 3: Heatmap showing the degree of preservation of colon consensus modules in three publicly available datasets. The x-axis describes the reference-test pair of datasets used for that respective column. The reference dataset is one of our two colon segments, where the connectivity profile from that dataset was evaluated for preservation in the test dataset. The y-axis names modules, which are grouped according to their inferred function. In the test dataset, gene module assignment was randomly permuted 200 times, and the mean and standard deviation from these permutations was used to convert connectivity statistics from the actual module assignment to a Z-score. A Z-score between 2 and 10 indicates “weak-to-moderate” support for the preservation of that module in the test network based on the profile of the reference network, while a Z-score above 10 indicates “strong” support. [file media-3.jpg]

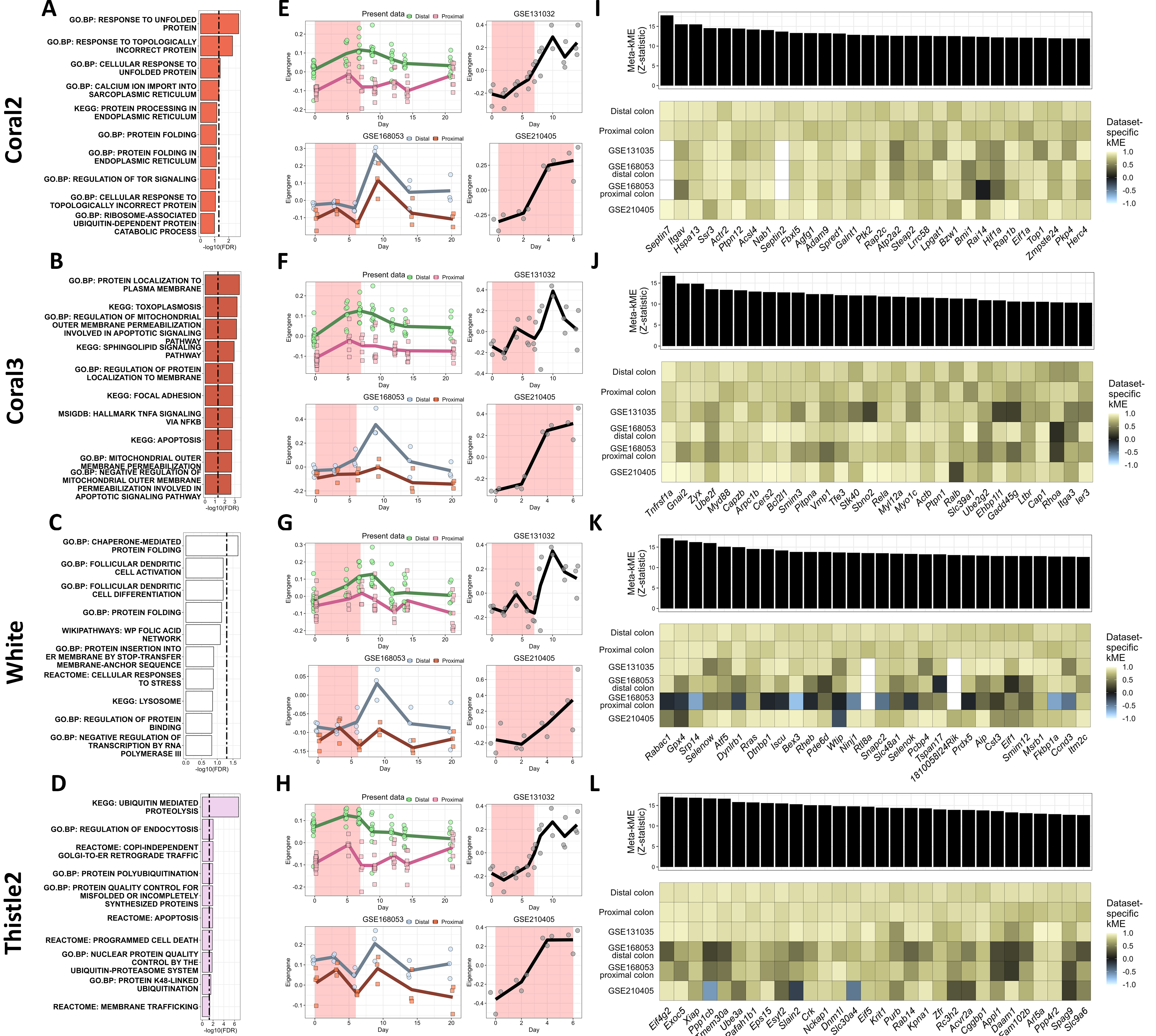

Supplement: Supplement 4 — Supp. Figure 4: Reproducible and experiment-specific stress responses in the intestine during DSS. A-D Gene set enrichment results from the ‘coral3,’ ‘coral2,’ ‘white,’ and ‘thistle2’ colon consensus modules. The top 10 pathways are shown, ranked on their unadjusted p-values. Bars show the −log10 transform of the FDR for that gene set. Bars reaching to the right of the dashed line reflect an FDR < 0.05. E-H Module eigengenes for each of four datasets: top-left shows our two datasets colored by colon segment; top-right shows GSE131032; bottom-left are the eigengenes from GSE168053 colored by colon segment; bottom-right shows GSE210405. The x-axis indicates day of the study, and the red shading indicates when DSS was administered. I-L Meta-module membership shown as a Z-statistic based on our two colon datasets with the corresponding dataset-specific module membership (correlation with the module eigengene, kME) for each gene displayed in a heatmap. The top 30 module members, defined by their high meta-kME, are shown here for each module. [file media-4.jpg]

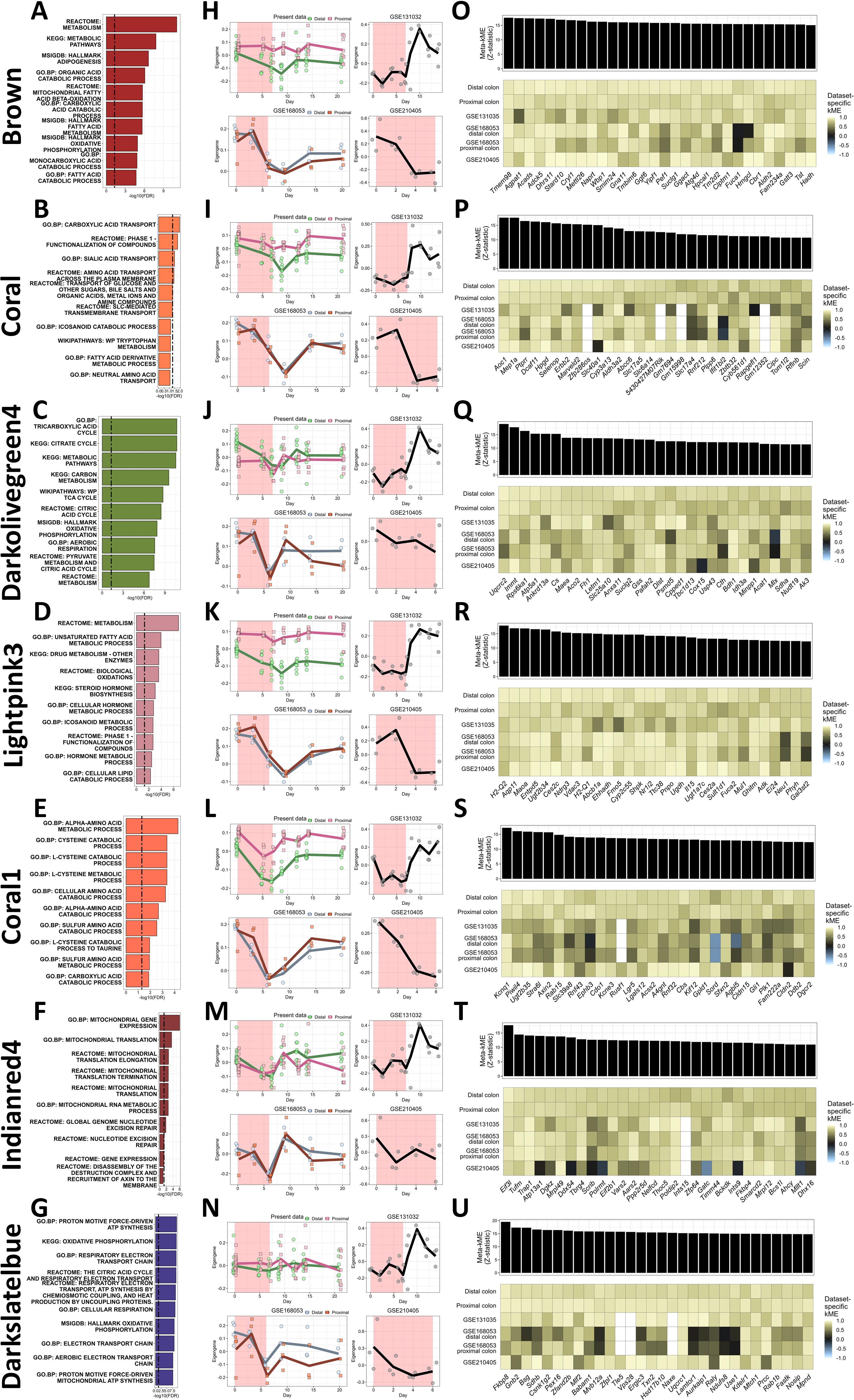

Supplement: Supplement 5 — Supp. Figure 5: Reproducible and study-specific metabolic disturbances in the colon during colitis. A-G Gene set enrichment results from the ‘brown,’ ‘coral,’ ‘darkolvegreen4,’ ‘lightpink3,’ ‘coral1,’ ‘indianred4,’ and ‘darkslateblue’ colon consensus modules. Gene sets are ranked by ascending p-value, and the top 10 pathways are displayed. Bars show the −log10 transform of the FDR for that gene set, and bars rising to the right of the dashed line reflect an FDR < 0.05. H-N Module eigengenes for each of four datasets: top-left shows our two datasets colored by colon segment; top-right shows GSE131032; bottom-left are the eigengenes from GSE168053 colored by colon segment; bottom-right shows GSE210405. The x-axis indicates day of the study, and the red shading indicates when DSS was administered. O-U Meta-module membership shown as a Z-statistic based on our two colon datasets with the corresponding dataset-specific module membership (correlation with the module eigengene, kME) for each gene displayed in a heatmap. The top 30 module members, defined by their high meta-kME, are shown here for each module. [file media-5.jpg]

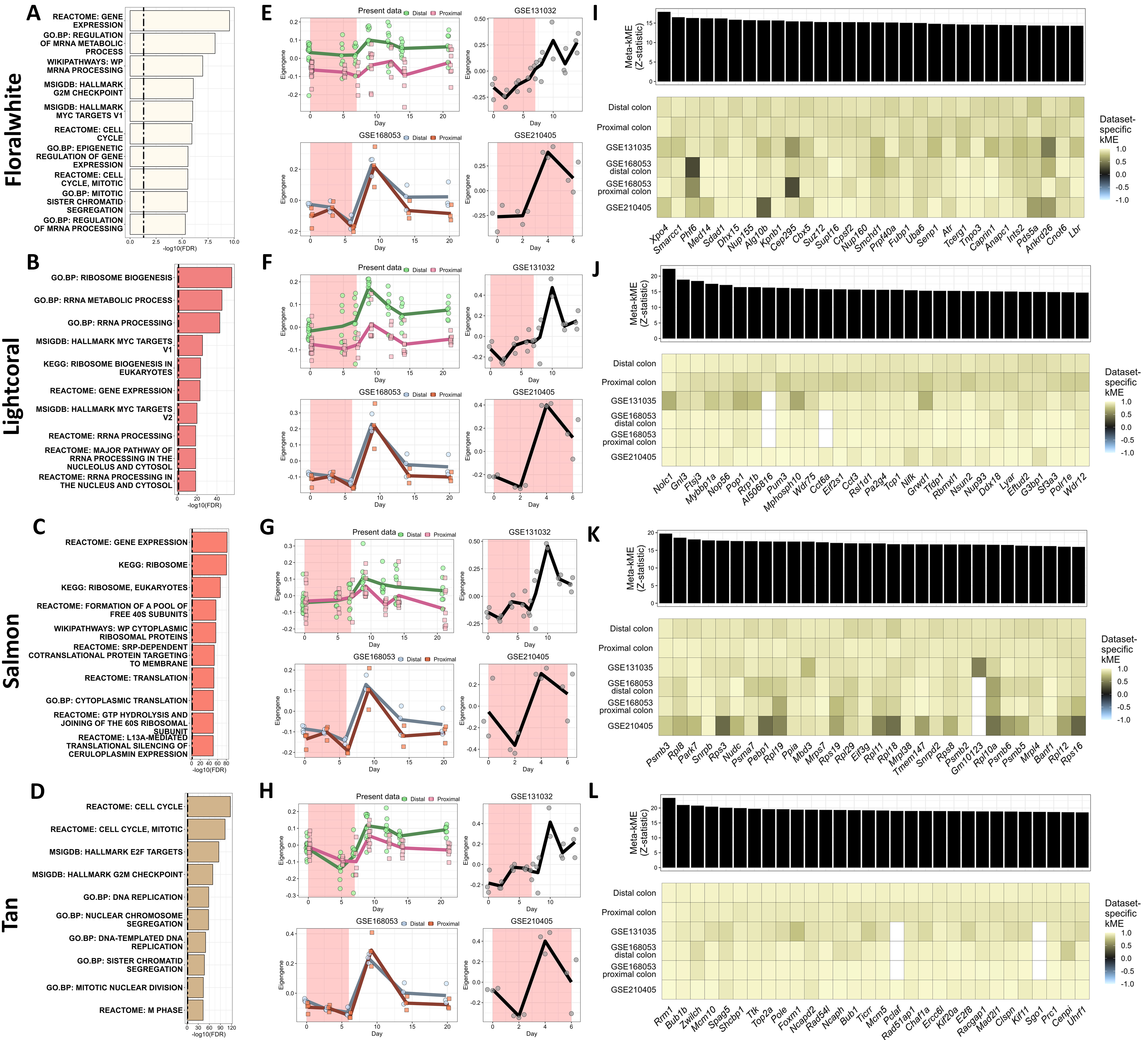

Supplement: Supplement 6 — Supp. Figure 6: Reproducible and study-specific intestinal repair processes during and after DSS-induced colitis. A-D Gene set enrichment results from the ‘floralwhite,’ ‘lightcoral,’ ‘salmon,’ and ‘tan’ colon consensus modules. The top 10 pathways are shown, ranked on their p-values. Bars show the −log10 transform of the FDR for that gene set, and bars rising to the right of the dashed line have an FDR < 0.05. E-H Module eigengenes for each of four datasets: top-left shows our two datasets colored by colon segment; top-right shows GSE131032; bottom-left are the eigengenes from GSE168053 colored by colon segment; bottom-right shows GSE210405. The x-axis indicates day of the study, and the red shading indicates when DSS was administered. I-L Meta-module membership shown as a Z-statistic based on our two colon datasets with the corresponding dataset-specific module membership (correlation with the module eigengene, kME) for each gene displayed in a heatmap. The top 30 module members, defined by their high meta-kME, are shown here for each module. [file media-6.jpg]

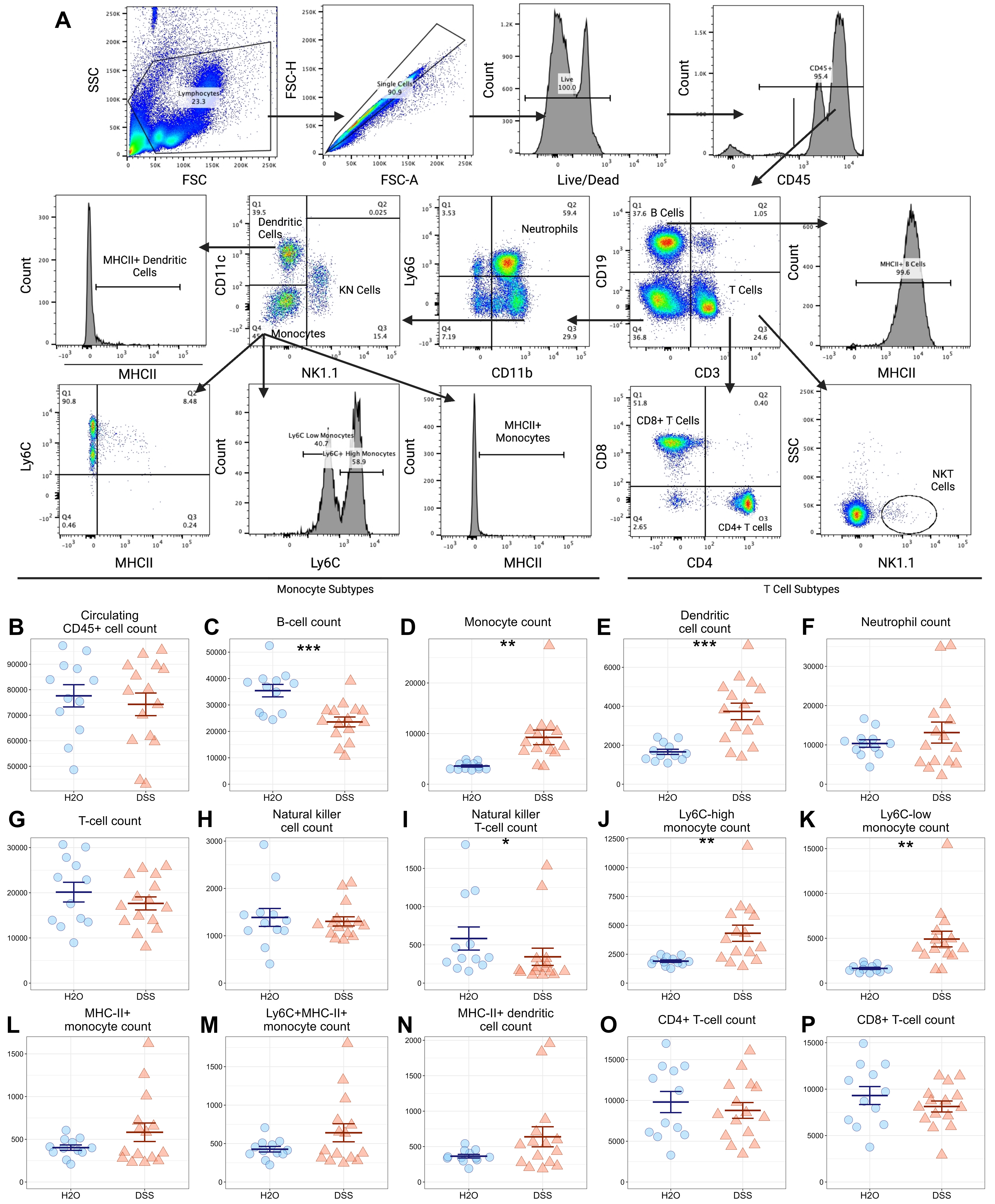

Supplement: Supplement 7 — Supp. Figure 7: Identification of peripheral immune cells with multi-color flow cytometry. A Gating strategy to identify circulating immune cells with flow cytometry. B Raw counts of CD45+ cells isolated from blood (Student’s t[25] = −0.530, p = 0.601). C Raw counts of circulating B-cells (Student’s t[25] = −4.000, p < 0.001). D Raw counts of circulating monocytes (Welch’s t[14.605] = 3.871, p = 0.002). E Raw counts of circulating dendritic cells (Welch’s t[16.692] = 4.671, p < 0.001). F Raw counts of neutrophils in circulation (Welch’s t[17.461] = 0.976, p = 0.342). G Counts of total T-cells in circulation (Student’s t[25] = −0.955, p = 0.351). H Counts of natural killer cells in circulation (Mann-Whitney U = 78, p = 0.575). I Counts of circulating natural killer T-cells (Mann-Whitney U = 39, p = 0.014). J Counts of Ly6C-high monocytes (Welch’s t[14.666] = 3.405, p = 0.004). K Counts of Ly6C-low monocytes (Welch’s t[14.496] = 3.714, p = 0.002). L Counts of MHC-II+ monocytes (Welch’s t[16.333] = 1.595, p = 0.130). M Counts of Ly6C+MHC-II+ monocytes (Welch’s t[16.604] = 1.742, p = 0.100). N Counts of MHC-II+ dendritic cells (Welch’s t[14.929] = 1.905, p = 0.076). O CD4+ T-cell counts (Student’s t[25] = −0.649, p = 0.522). P CD8+ T-cell counts (Student’s t[25] = −1.085, p = 0.288). Data in B-P are presented as mean ± SEM. *p < 0.05, **p < 0.01, ***p < 0.001. [file media-7.jpg]

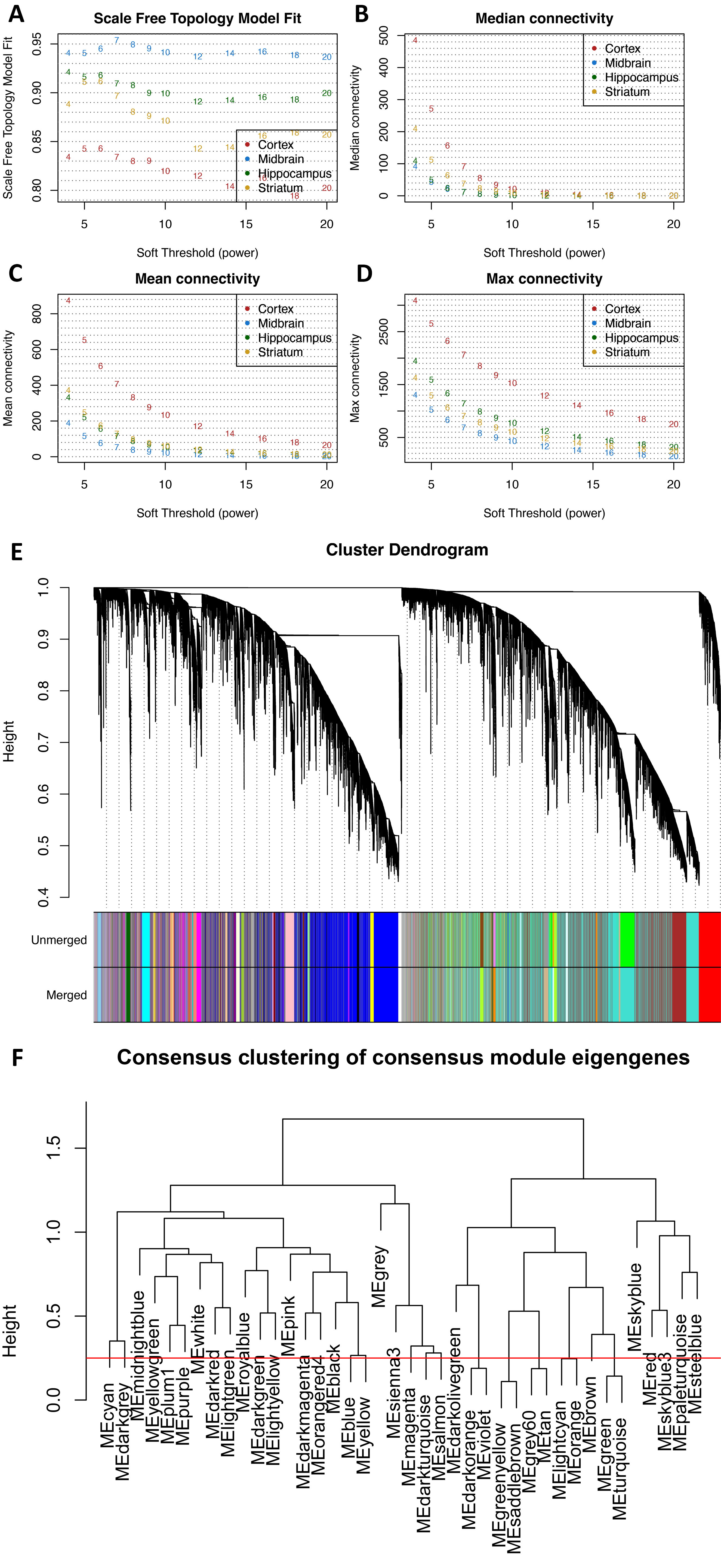

Supplement: Supplement 8 — Supp. Figure 8: Consensus co-expression network construction in four brain regions. A Scale-free topology model R2 in cortex (red), midbrain (blue), hippocampus (green), and striatum (yellow) RNA sequencing datasets as a function of soft thresholding/beta power. The median (B), average (C), and maximum (D) connectivity of both segments are shown as functions of soft thresholding power. From these data, a soft power of 6 was selected to maximize the model fit, which was used as the primary criterion of soft power selection due to the discrepancies in connectivity profiles between regions. For example, notice that the cortex dataset displays much higher connectivity than the other three regions at all soft powers examined. E Gene dendrogram with original (unmerged) and merged module labels. Modules were merged based on average linkage distance shown in the dendrogram in F. [file media-8.jpg]

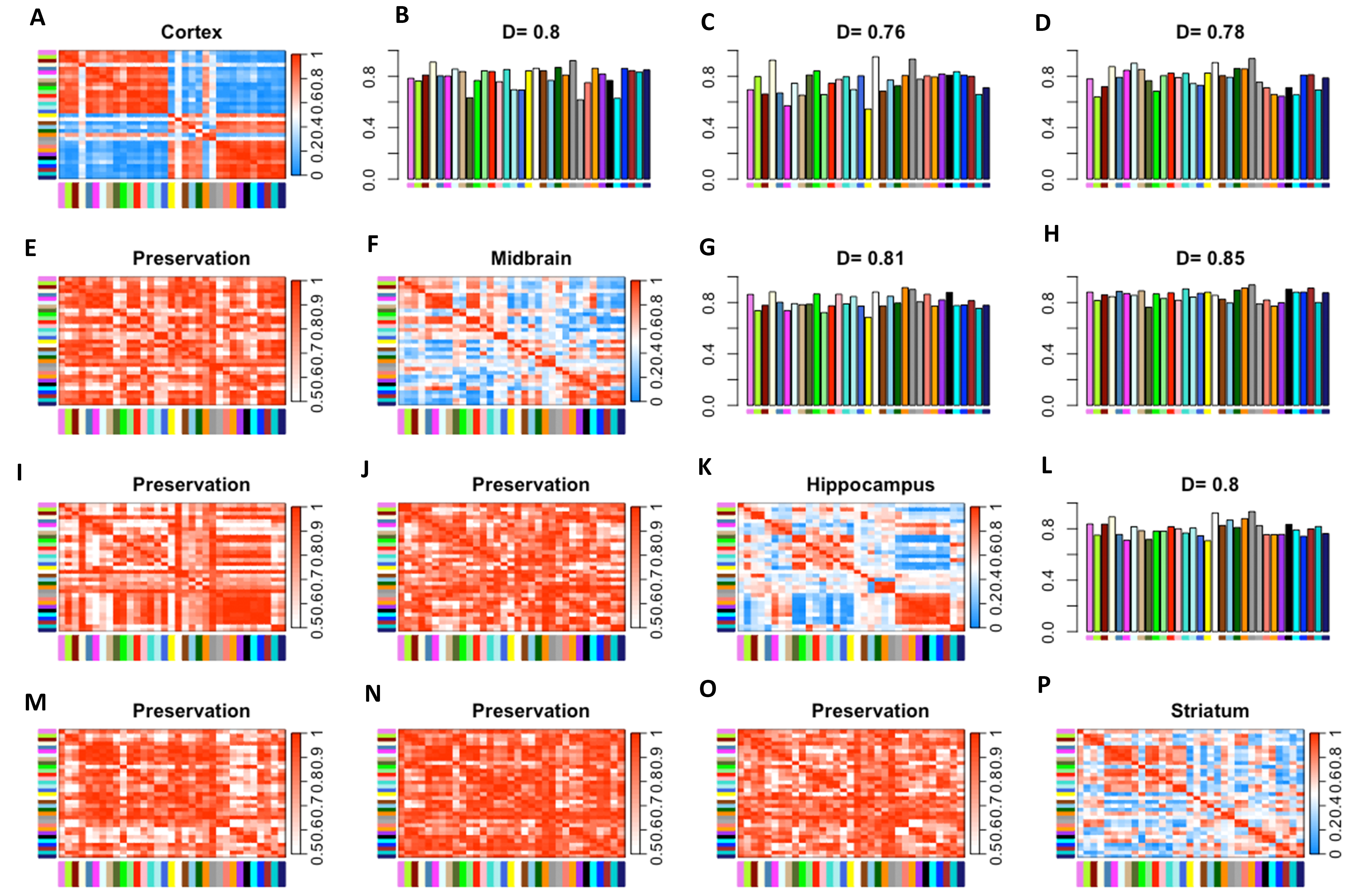

Supplement: Supplement 9 — Supp. Figure 9: Assessment of the preservation of eigengene adjacency between cortex, midbrain, hippocampus, and striatum. A Intermodular correlation heatmap in cortex samples. Each row and column correspond to the eigengene of a module. Red represents a positive correlation, while blue represents a negative correlation. Bar chart showing the aggregate preservation of individual eigengenes between cortex and midbrain samples (B), between cortex and hippocampus (C), and between cortex and striatum (D). E Heatmap displaying the pairwise preservation of modules, defined as one minus the absolute value of the difference in the eigengene adjacencies, in cortex and midbrain samples. F Intermodular correlation heatmap in midbrain samples, coded as in A. Bar chart showing the aggregate preservation of individual eigengenes between midbrain and hippocampus (G) and between midbrain and striatum (H) samples. Heatmap displaying the pairwise preservation of modules between hippocampus and cortex (I) and between hippocampus and midbrain (J), coded as in E. K Intermodular correlation heatmap in hippocampus samples, coded as in A. L Bar chart showing the aggregate preservation of individual eigengenes in hippocampus and striatum samples. Heatmap showing the pairwise preservation of modules between cortex and striatum (M), between midbrain and striatum (N), and between hippocampus and striatum (O), coded as in E. P Intermodular correlation heatmap in striatum samples, coded as in A. In short, the diagonal of this matrix shows intermodular correlations in region-specific datasets. Red indicates a positive correlation between eigengenes while blue indicates negative. The upper triangle contains bar charts of eigengene preservation between pairs of datasets and the lower triangle shows heatmaps of the pairwise preservation networks, where “preservation” is calculated as one minus the absolute value of the difference of the eigengene correlations between the two networks. A preservation [file media-9.jpg]

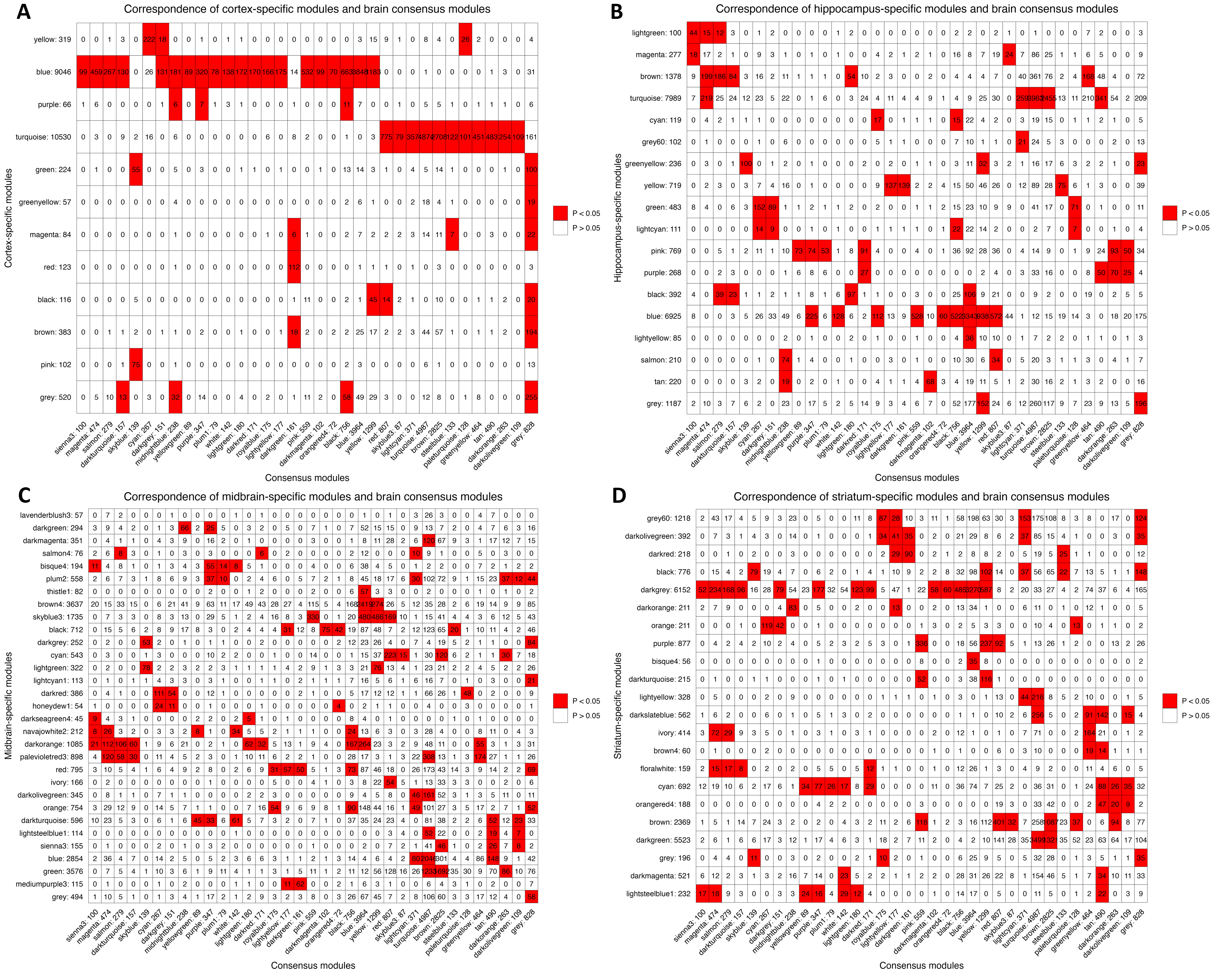

Supplement: Supplement 10 — Supp. Figure 10: Assessment of the preservation of gene module assignment in cortex, hippocampus, midbrain, and striatum. Multiple Fisher’s exact test examining the independence of gene assignment to consensus modules and cortex-specific modules (A), hippocampus-specific modules (B), midbrain-specific modules (C), and striatum-specific modules (D). Columns are consensus modules and rows are region-specific modules. Cells shaded red indicate a significant Fisher’s exact test (p < 0.05) after Bonferroni’s correction, meaning there is significant overlap between the region-specific module and the consensus module. The number in each cell reflects the number of genes overlapping the two modules shown at that intersection. [file media-10.jpg]

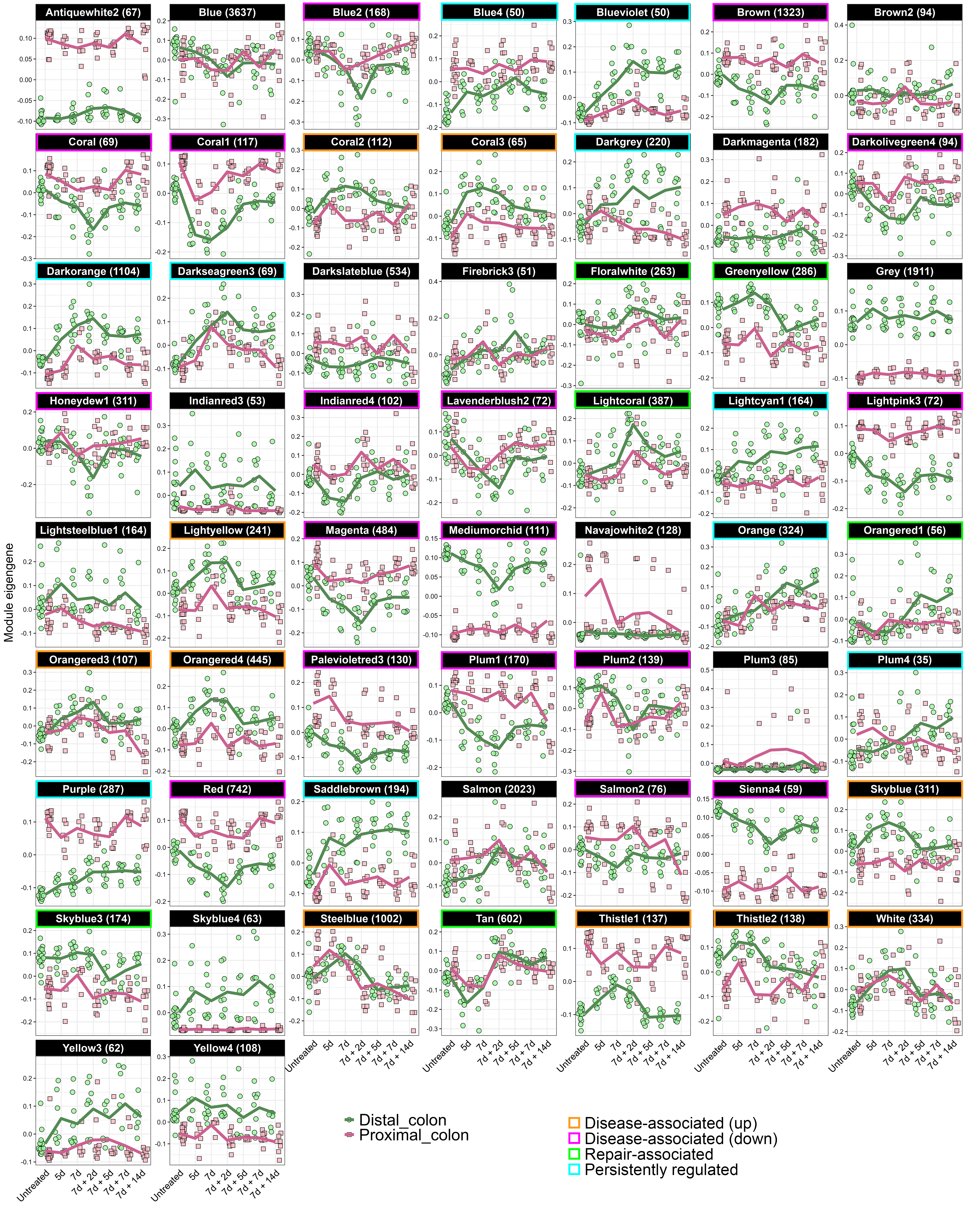

Supplement: Supplement 11 — Supp. Figure 11: Classification of colon consensus modules based on eigengenes. Eigengene plots for every colon consensus module identified by our analysis. The color name of the module is shown in the black title box, and the number of genes assigned to that module is shown in parentheses next to it. Distal colon sample eigengenes are shown by the green circles, and proximal colon eigengenes are shown by the pink squares. Module categorization is shown by the colored boxes around the title box: orange corresponds to modules up-regulated during disease, magenta corresponds to modules down-regulated during disease, green corresponds to modules regulated during the repair phase, and cyan corresponds to modules that appear to be persistently regulated throughout the dosing scheme. [file media-11.jpg]

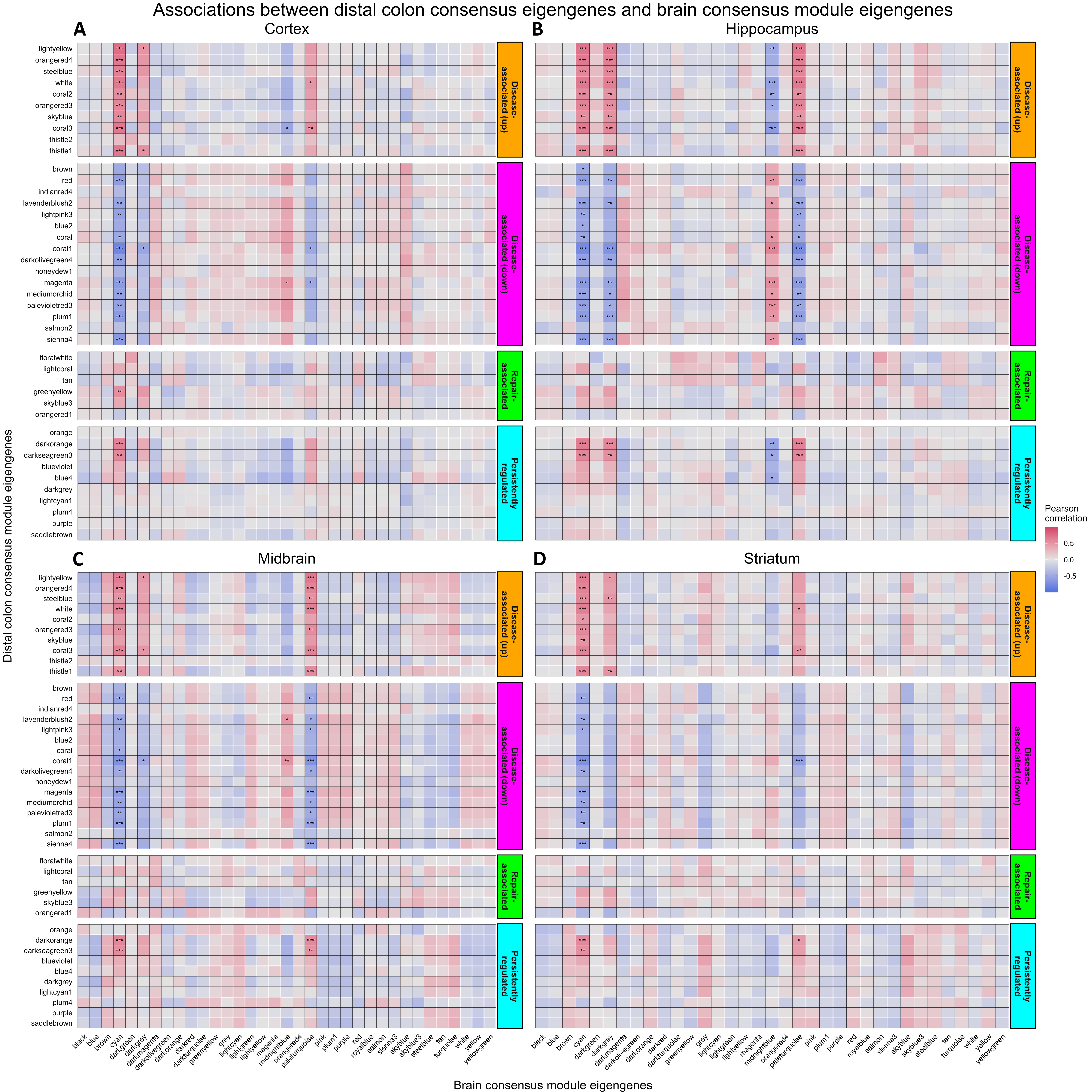

Supplement: Supplement 12 — Supp. Figure 12: Transcriptomic disturbances in brain during colitis are associated with disease-associated gene programs in the intestine. Heatmaps showing the Pearson correlation coefficient between colon consensus module eigengenes in distal colon (rows) and brain consensus module eigengenes in cortex (A), hippocampus (B), midbrain (C), and striatum (D). Red indicates a positive correlation while blue indicates a negative correlation. Numbers in each cell reflect the p-value of the correlation after Bonferroni’s correction, rounded to two digits. Distal colon module eigengenes are separated according to their expression profiles outlined in Fig. SF2 and Fig. 2. Note the number of significant correlations between the brain ‘paleturquoise’, ‘midnightblue’, ‘cyan’, and ‘darkgrey’ modules with distal colon modules that are differentially regulated preferentially during DSS administration compared to the paucity of significant correlations between those four modules with distal colon modules that are differentially regulated during the repair phase or consistently throughout the paradigm. [file media-12.jpg]

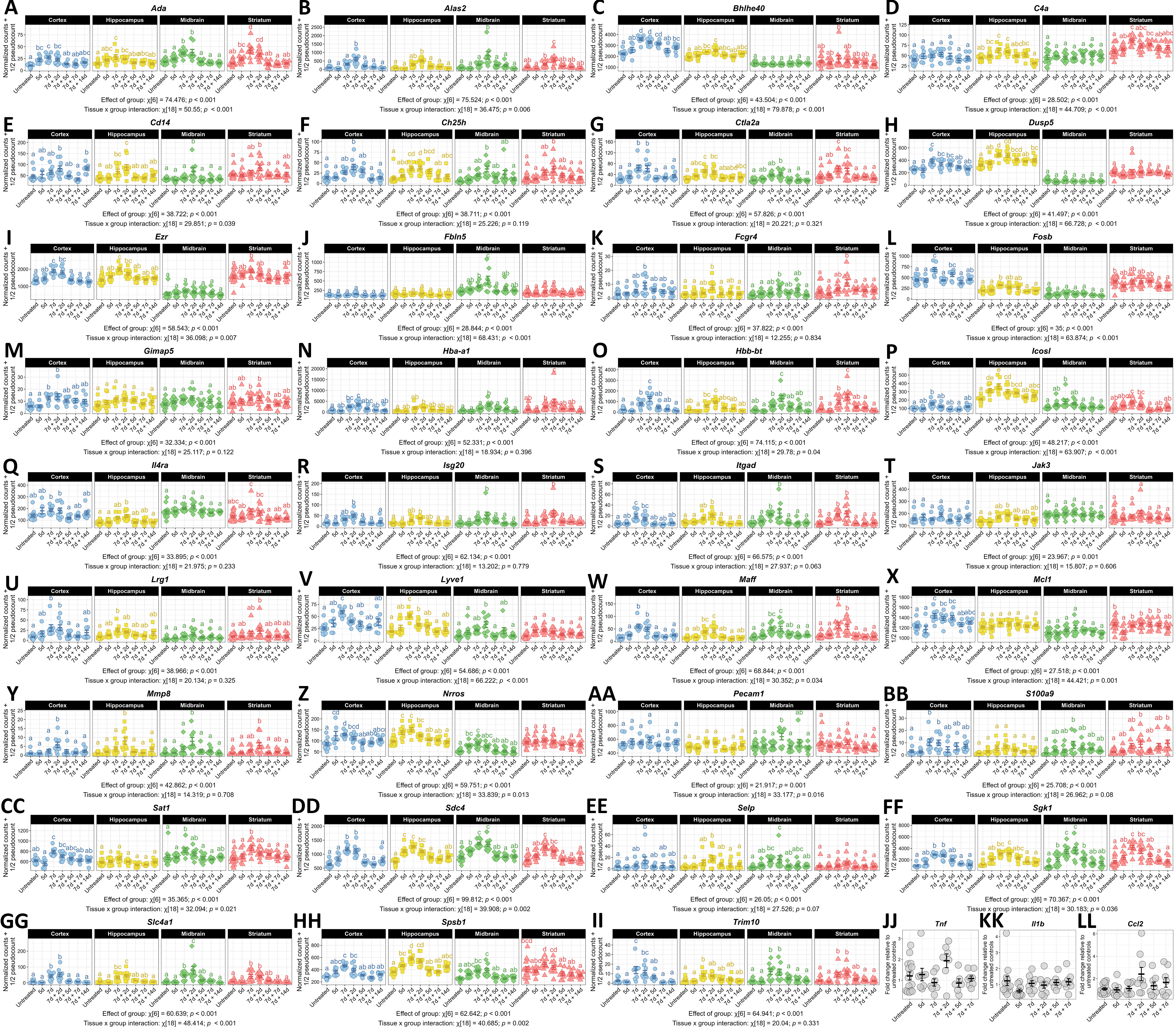

Supplement: Supplement 13 — Supp. Figure 13: DSS-associated differential expression of immune system genes in the brain. DESeq2-normalized counts for genes related to immune system processes are shown in A-GG. Separate facets were created for each brain region, where blue circles correspond to cortex samples, yellow squares correspond to hippocampus samples, green diamonds correspond to midbrain samples, and red triangles correspond to striatum samples. Statistical results are shown with the plots, gathered from the multiple mixed-effects modeling procedure described in the Methods section. Pairwise comparisons were made within each region, and the compact letter display (CLD) is shown. Groups that share a letter within each facet are not statistically significantly different (p > 0.05 after Tukey’s correction) from each other. The expression of Tnf (one-way ANOVA: F[5, 45] = 2.290, p = 0.062) (HH), Il1b (one-way ANOVA: F[5, 48] = 1.137, p = 0.354) (II), and Ccl2 (one-way ANOVA: Welch’s F[5, 19.652] = 1.294, p = 0.306) (JJ) was measured with qPCR in midbrain samples from all groups excluding the 7d DSS + 14d H2O group. All data in this figure are shown as mean ± SEM. [file media-13.jpg]

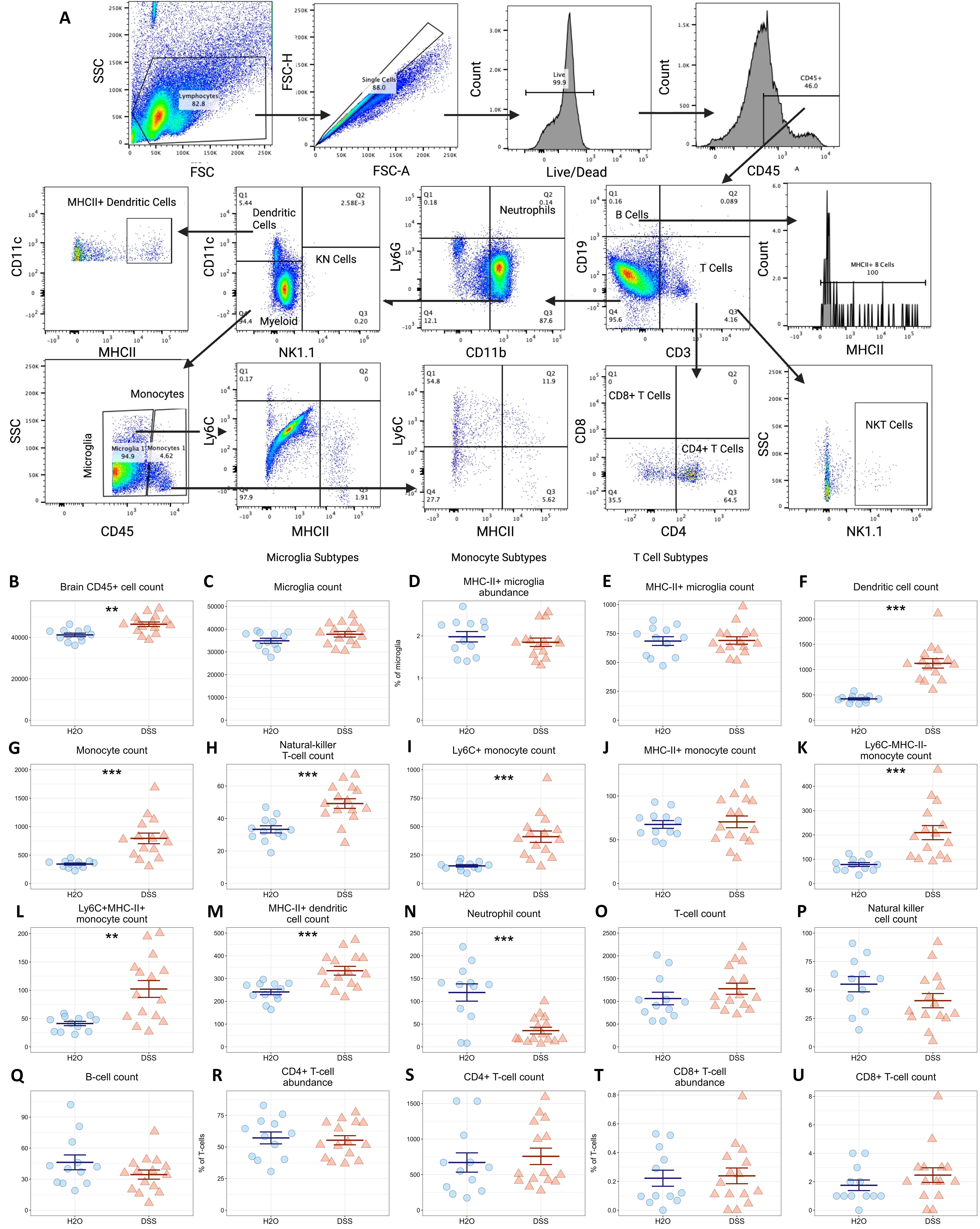

Supplement: Supplement 14 — Supp. Figure 14: Identification of microglia and other immune cell populations in brain by multi-color flow cytometry. A The gating strategy to identify microglia and other immune cell populations in the brain with flow cytometry. B Raw counts of CD45+ cells in the brain (Student’s t[25] = 3.522, p = 0.002). C Raw counts of microglial cells in the brain (Student’s t[25] = 1.683, p = 0.105). D MHC-II+ microglia abundance expressed as a percentage of total microglia (Student’s t[25] = −0.840, p = 0.409). E Raw counts of MHC-II+ microglia (Student’s t[25] = 0.102, p = 0.919). F Counts of dendritic cells in brain (Welch’s t[15.317] = 7.341, p < 0.001). G Total monocyte counts in brain (Welch’s t[15.050] = 4.761, p < 0.001). H Natural killer T-cell counts in brain (Student’s t[25] = 4.117, p < 0.001). I Ly6C+ monocyte subtype counts (Welch’s t[15.172], p < 0.001). J MHC-II+ monocyte subset counts (Welch’s t[23.256] = 0.370, p = 0.715). K Counts of Ly6C-MHC-II-monocytes in brain (Welch’s t[16.007] = 4.361, p < 0.001). L Counts of Ly6C+MHC-II+ double-positive monocytes in brain (Welch’s t[15.802] = 3.981, p = 0.001). M Counts of MHC-II+ dendritic cells (). N Counts of neutrophils (Welch’s t[14.249] = −4.116, p = 0.001). O Total T-cell counts (Mann-Whitney U = 62, p = 0.183). P Natural killer cell counts (Student’s t[25] = −1.573, p = 0.128). Q B-cell counts (Mann-Whitney U = 66, p = 0.251). R CD4+ T-cell abundance as a percentage of total T-cells. S CD4+ T-cell counts (Mann-Whitney U = 81, p = 0.683). T CD8+ T-cell abundance as a percentage of total T-cells. Data from R and T were analyzed with a two-way ANOVA due to the interdependence of relative abundance of subtypes (main effect of DSS – F[1, 25] = 0.099, p = 0.756; main effect of subtype – F[1, 25] = 362.027, p < 0.001; interaction – F[1, 25] = 0.099, p = 0.756). U CD8+ T-cell counts (Mann-Whitney U = 69, p = 0.305). We note these counts are extremely low and are likely not reliable. Data in B-U are presented as mean [file media-14.jpg]
